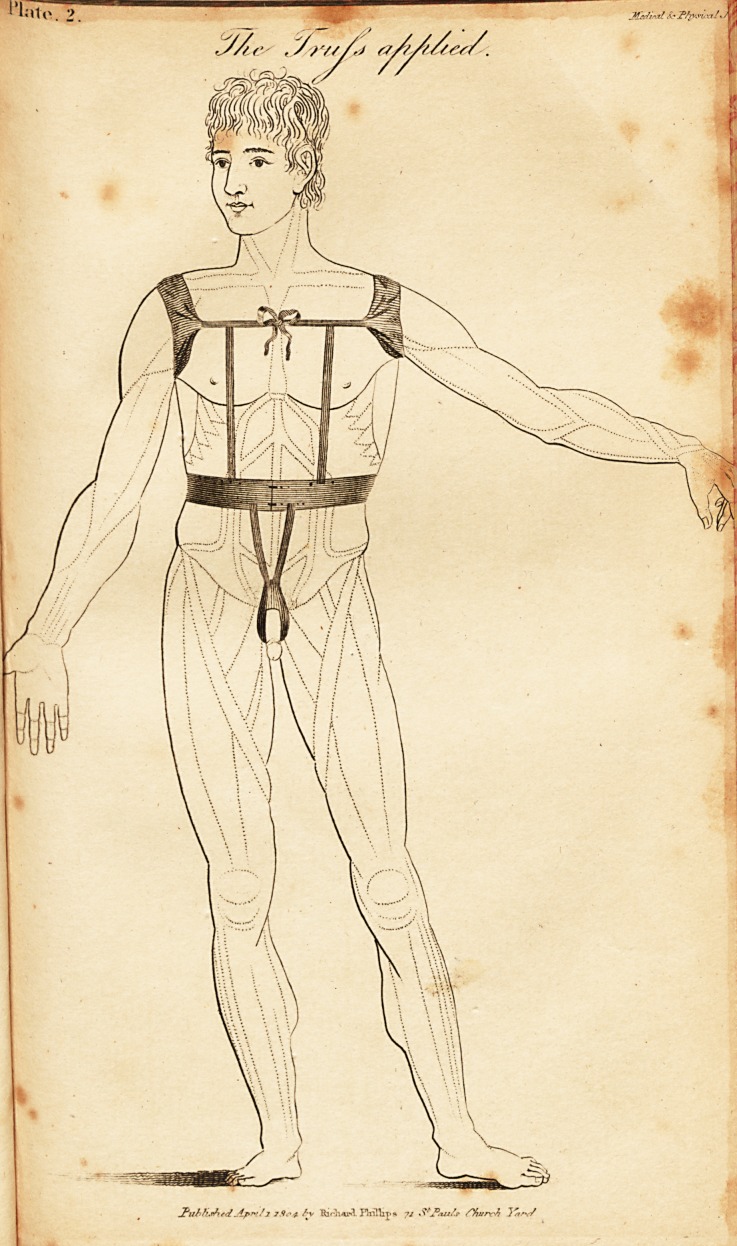# Mr. Luxmoore's Improved Bandage

**Published:** 1804-04-01

**Authors:** Thomas Luxmoore

**Affiliations:** St. Mary Axe


					304
Mr. Luxmoore's improved Bandage.
To the Editors of the Medical and Phyfical Journal.
Gentlemen,
The Bandage called the Bag Truss having been gene-
rally complained of, as inadequate to the purposes for
which it is applied, I beg leave to present you with a
Drawing of one of my own construction, which effectually
suspends the scrotum, without being liable to displacement
by the most violent exercise.
I am, See.
THOMAS LUXMOOKE.
St. Mary Arc,
Jan. 21, 1804.
Plate I. represents the Bandage.
AA. The arm holes.
B. A strap passing behind the shoulders.
CC. Two straps to tie it across the breast.
DD. Four straps, connecting the upper and lower part
of the bandage; two behind and two before.
E'. Passes round the body, above the hips, and is fasten-
' ed with hooks before.
R Straps beneath the thighs.
H. The bag itself.
Plate II. The Bandage applied.
As
~ Jit Jica! Sc^.W-'tca/ Journa
Volxip&i
JM'-tJi.-.xl trA/j
JPubh.rAtdjtflt1 7.9^4. PiriHlp? J1 S*J?ait/p <^Vturrh J/?/*y/
, /? '/ / /j &/>// //?'/.

				

## Figures and Tables

**Plate 1 f1:**
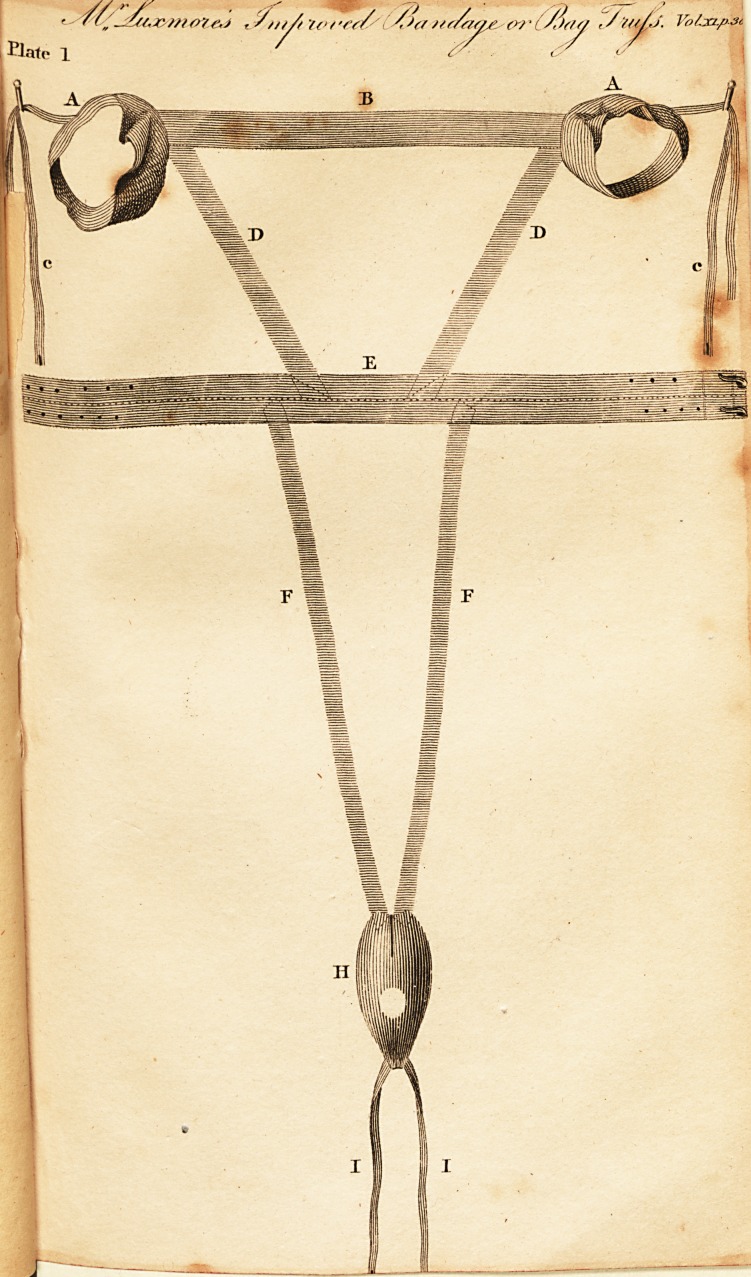


**Plate. 2. f2:**